# Omega-3 and omega-6 polyunsaturated fatty acids and their potential therapeutic role in protozoan infections

**DOI:** 10.3389/fimmu.2024.1339470

**Published:** 2024-04-03

**Authors:** Sajid Ur Rahman, Tzu-Nin Weng, Abdul Qadeer, Saqib Nawaz, Hanif Ullah, Chien-Chin Chen

**Affiliations:** ^1^ School of Agriculture and Biology, Shanghai Jiao Tong University, Shanghai, China; ^2^ Shanghai Veterinary Research Institute, Chinese Academy of Agricultural Sciences, Shanghai, China; ^3^ College of Animal Science and Technology, Anhui Agricultural University, Hefei, China; ^4^ Department of Stomatology, Ditmanson Medical Foundation Chia-Yi Christian Hospital, Chiayi, Taiwan; ^5^ Department of Cell Biology, School of Life Sciences, Central South University, Changsha, China; ^6^ West China Hospital, School of Nursing, Sichuan University, Chengdu, China; ^7^ Department of Pathology, Ditmanson Medical Foundation Chia-Yi Christian Hospital, Chiayi, Taiwan; ^8^ Department of Cosmetic Science, Chia Nan University of Pharmacy and Science, Tainan, Taiwan; ^9^ Department of Biotechnology and Bioindustry Sciences, College of Bioscience and Biotechnology, National Cheng Kung University, Tainan, Taiwan; ^10^ Doctoral Program in Translational Medicine, Rong Hsing Research Center for Translational Medicine, National Chung Hsing University, Taichung, Taiwan

**Keywords:** omega polyunsaturated fatty acids, prevention, parasitic infections, mechanism of action, supportive therapy

## Abstract

Protozoa exert a serious global threat of growing concern to human, and animal, and there is a need for the advancement of novel therapeutic strategies to effectively treat or mitigate the impact of associated diseases. Omega polyunsaturated fatty acids (ω-PUFAs), including Omega-3 (ω-3) and omega-6 (ω-6), are constituents derived from various natural sources, have gained significant attention for their therapeutic role in parasitic infections and a variety of essential structural and regulatory functions in animals and humans. Both ω-3 and ω-6 decrease the growth and survival rate of parasites through metabolized anti-inflammatory mediators, such as lipoxins, resolvins, and protectins, and have both *in vivo* and *in vitro* protective effects against various protozoan infections. The ω-PUFAs have been shown to modulate the host immune response by a commonly known mechanism such as (inhibition of arachidonic acid (AA) metabolic process, production of anti-inflammatory mediators, modification of intracellular lipids, and activation of the nuclear receptor), and promotion of a shift towards a more effective immune defense against parasitic invaders by regulation the inflammation like prostaglandins, leukotrienes, thromboxane, are involved in controlling the inflammatory reaction. The immune modulation may involve reducing inflammation, enhancing phagocytosis, and suppressing parasitic virulence factors. The unique properties of ω-PUFAs could prevent protozoan infections, representing an important area of study. This review explores the clinical impact of ω-PUFAs against some protozoan infections, elucidating possible mechanisms of action and supportive therapy for preventing various parasitic infections in humans and animals, such as toxoplasmosis, malaria, coccidiosis, and chagas disease. ω-PUFAs show promise as a therapeutic approach for parasitic infections due to their direct anti-parasitic effects and their ability to modulate the host immune response. Additionally, we discuss current treatment options and suggest perspectives for future studies. This could potentially provide an alternative or supplementary treatment option for these complex global health problems.

## Introduction

Omega-3 and omega-6 polyunsaturated fatty acids are synthesized from the essential fatty acids alpha-linolenic acid and linoleic acid, respectively. They are fundamental components of living cells and have been found to significantly contribute to the prevention and treatment of a variety of health problems (1).

PUFAs can be divided into two primary groups: ω-3 PUFAs and ω-6 PUFAs, with their main distinction lying in the positioning of double bonds along the carbon chain. ω-6 PUFAs have their initial double bonds starting at the sixth carbon atom, whereas ω-3 PUFAs begin at the third carbon atom, measured from the methyl end of the carbon chain, which is referred to as the ω-carbon (2). Linoleic acid (LA) (18:2 ω-6) and arachidonic acid (AA) (20:4 ω-6) are the two most common ω-6 PUFAs in diets. Western diets are high in ω-6 PUFAs and low in ω-3 PUFAs, resulting in a high ω-6/ω-3 ratio of up to 20-30 (3). The three primary ω-3 PUFAs are α-linolenic acid (18:3 ω-3), eicosapentaenoic acid (20:5 ω-3), and docosahexaenoic acid (DHA, 22:6 ω-3). It is important to distinguish between α-LA (an ω-3 precursor) and γ-linolenic acid (GLA), which is 18:3 but belongs to the ω-6 fatty acid series. LA (precursor to ω-6 fatty acids) and α-LA (precursor to ω-3 fatty acids) are essential fatty acids, as mammals cannot synthesize them (2). Considering the adverse effects of synthetic medications, there is a growing trend in the utilization of natural therapies and supplements in the contemporary medical landscape (1).

**Figure 1:**
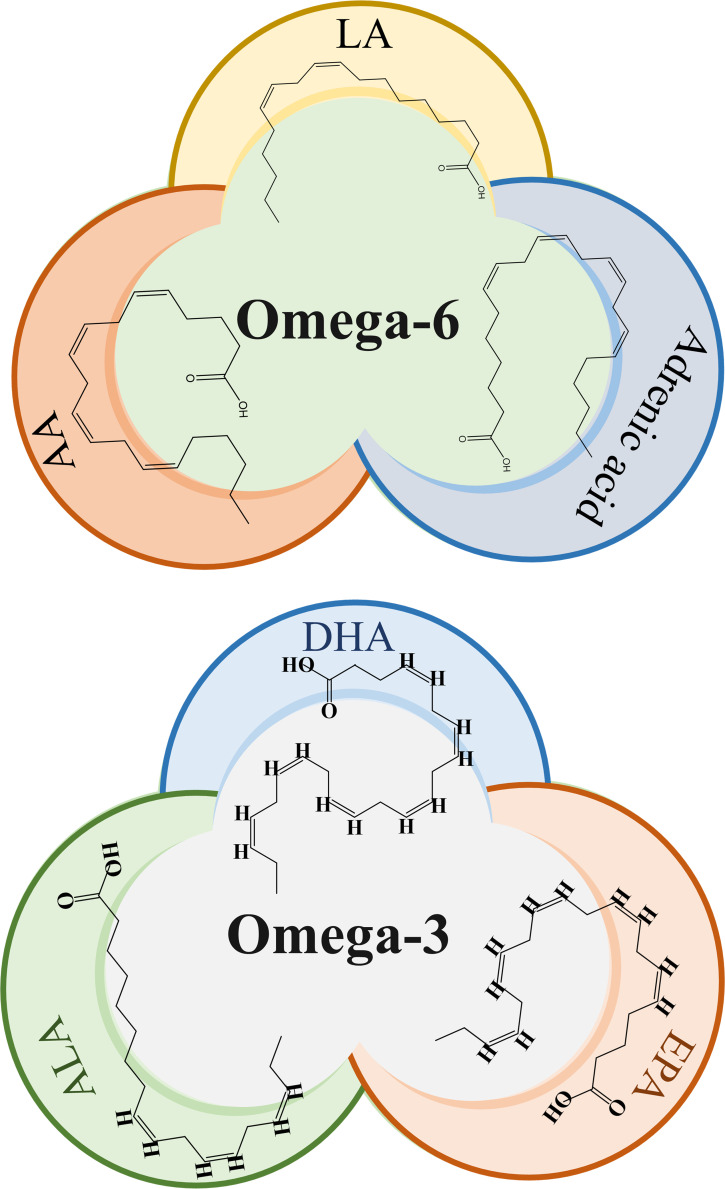
Chemical structure of omega-3 and omega-6 PUFAs. LA, Linoleic acid; AA, Arachidonic acid, ALA, α-linolenic acid; EPA, eicosapentaenoic acid; DHA, docosahexaenoic acid.

ω-3 fatty acids such as Eicosapentaenoic acid (EPA), DHA, and Alpha-linolenic acid (ALA), as well as ω-6 fatty acids like LA and AA as shown in **Figure 1**, are important structural components of the cell membrane. They serve as precursors to bioactive lipid mediators and provide a source of energy. These PUFAs are components of the human and animal diet and are highly regarded as dietary supplements. Numerous studies have previously demonstrated the potential benefits of ω-PUFAs in increasing nutritional consumption, including cardiovascular (4), neurodegenerative (5), inflammatory diseases (6), as well as for some cancer types, mostly prostatic, colorectal, and mammary cancer (7, 8). Concurrently, a considerable number of *in vitro* and *in vivo* studies have consistently shown the importance of ω-PUFAs as protective and therapeutic agents against cardiac arrhythmias, hypertriglyceridemia, and inflammation (9).

Besides their excellent functions in treating innate immune disorders such as allergies or atopic dermatitis, type 1 diabetes (T1D), rheumatoid arthritis (RA), systemic Lupus erythematosus (SLE), and multiple sclerosis (10). ω-PUFAs also showed good potential to prevent various exogenous pathogens, including several kinds of parasites. A recent study has shown that DHA, a representative of ω-3, strongly persuaded autophagy in murine bone marrow-derived macrophages, which may prevent *T. gondii* infection (11). These PUFAs enhanced recovery and decreased the risk of infection (12). Bioactive derivatives of PUFAs regulate inflammation by influencing the production of molecules such as prostaglandins, leukotrienes, and thromboxane. In addition, specialized pro-resolving mediators (SPMs) like resolvins, protectins, and maresins play an important role in controlling inflammatory responses (13). These SPMs promote anti-inflammatory and pro-resolution properties while maintaining immune function, thereby restoring homeostasis. They accomplish this by downregulating pro-inflammatory cytokines and upregulating anti-inflammatory cytokines. SPMs also help phagocytic cells clear cellular debris and lower oxylipin levels, which promote inflammation. The synthesis of these bioactive mediators from PUFAs such as DHA and EPA involve enzymes such as phospholipases A2 (PLA2s), cyclooxygenases (COX), lipoxygenases (LOX), and cytochrome P450 (CYP450). These mechanisms include inhibiting the metabolism of AA, producing anti-inflammatory mediators, modifying intracellular lipids, and activating nuclear receptors (13, 14). Here, we summarize the sources of ω-3 and ω-6 PUFAs and the current understanding of the therapeutic role of ω-PUFAs in the prevention of various protozoan infections. Moreover, we discuss the current treatment options and suggest future research directions.

## Challenges in parasitic diseases and the need for therapeutic progress

Infectious diseases exert significant health impacts on both humans and livestock. The parasite residing in the gut can change the composition of the microbiome, and these changes can have significant effects on gut homeostasis and host immunity (15). Many of these diseases are caused by different parasites. Amongst others, these comprise the causative agents of malaria, coccidiosis, trypanosomiasis, toxoplasmosis, schistosomiasis, etc. Every year, these parasites cause a high rate of mortality and morbidity in endemic countries (16, 17). Recent studies have reported alarming figures, with parasitic infections contributing to a significant percentage of health losses. For instance, in regions affected by malaria, the World Health Organization (WHO) estimated 619,000 malaria deaths globally in 2021 compared to 625,000 in the first year of the pandemic. In 2019, before the pandemic struck, the number of deaths stood at 568,000. The global tally of malaria cases reached 247 million in 2021 compared to 245 million in 2020 and 232 million in 2019 (18).

Similarly, 54.3, 31.7, 70.9, and 52.9% mortality rates associated with the *Eimeria* parasite have been reported in Turkey, India, Ethiopia, and Nigeria, respectively (19). Meanwhile, Chagas disease affects about 7 million people worldwide (20). These figures underscore the severity of parasitic infections and highlight the urgent need for novel therapeutic agents against these parasites. Meanwhile, vaccines and successful and safe treatment are still deficient, and most of the drugs used were shown drug-resistant. Therefore, novel therapeutic agents against these parasites are urgently needed. Currently, the situation is very serious because in low-income countries the pharmaceutical industries are unable to develop new drugs against these infectious agents. Thus, natural products signify some good opportunities to discover novel therapeutic molecules (16, 17). On the other hand, the occurrence of partial immunity after exposure suggests the potential for a successful and effective vaccine, yet the identification of exact markers for protection remains a complex task (21). The control of parasitic infections needs great attention in the fields of public health, parasitology research, medical science, and political will. However, during the entire spectrum of parasitic diseases of animals and humans, there is an urgent need for better treatment and the search for the best and novel drugs. Recently, natural products have become a beneficial source of treatments for clinical and preventive use, and ω-PUFAs are such kind of attractive components that have gained great attention in clinical research (22).

## Sources of omega PUFAs

The consumption of ω-3 PUFAs is generally lacking because of insufficient sources; however, western food is typically the high source of ω-6 PUFAs (23). Most vegetable oils and seeds such as sunflower, corn, wheat, grape seed, rapeseed, poppy seed, palm, hemp, cottonseed, and soybean are rich in ω-6 PUFAs in the form of LA, however, a low proportion of ω-3 ALA. The ALA is frequently found in green leafy vegetables, walnuts, flaxseed, canola oils, and soybeans. However, DHA and EPA are found in fish oils, such as mackerel, salmon, anchovies, trout, sardines, and algae (24–26). AA is a nutritional ω-6 supplement mostly found in poultry, eggs, and meaty organs, while gamma-linolenic acid (GLA) is frequently available in borage oil, evening primrose oil, and rich amounts in human milk. In animal and plant-based diets, these PUFAs originate in different forms such as phospholipids, triacylglycerols, cholesterol esters, and diacylglycerols (27). The animal and plant-based ω-PUFAs sources and the obtained proportion are listed in **Table 1**.

**Table 1:** The proportion of ω-PUFAs (g/100g) in various animal and plant-derived sources.

Sources	ω-3 PUFAs			ω-6 PUFAs			References
	ALA	DHA	EPA	LA	ADA	DPA	
Salmon	–	18.23	13.3	–	–	2.99	(28)
Herring	–	4.21	6.28	–	–	0.62	
Sardine	–	10.67	10.15	–	–	1.98	
Sunflower	0.33	–	–	49.90	–	–	(29)
Soybean	7.6	–	–	51.36	–	–	
Corn	0.6	–	–	49.83	–	–	
Wheat	5.3	–	–	55	–	–	
Almond	0.3	–	–	10.5	–	–	(30, 31)
Walnuts	6.6	–	–	34	–	–	
Hazelnuts	0.11	–	–	5.1	–	–	
Lettuce	0.15	–	–	0.06	–	–	(31)
Green broccoli	0.11	–	–	0.03	–	–	
Brussels sprouts	0.17	–	–	0.08	–	–	
Beef	0.08	–	–	0.13	–	–	(31)
Pork, lean meat	–	–	–	1.63	0.03	–	
Lamb	0.11	–	–	0.11	–	–	

ALA, α-linolenic acid; DHA, docosahexaenoic acid; EPA, eicosapentaenoic acid LA, Linoleic acid; ADA, Adrenic acid; AA, Arachidonic acid.

The above data were obtained from https://fdc.nal.usda.gov and http://www.bda-ieo.it/wordpress/en/?page_id=23.

## Parasitic diseases and their available treatments

### Toxoplasmosis

**Figure 2:**
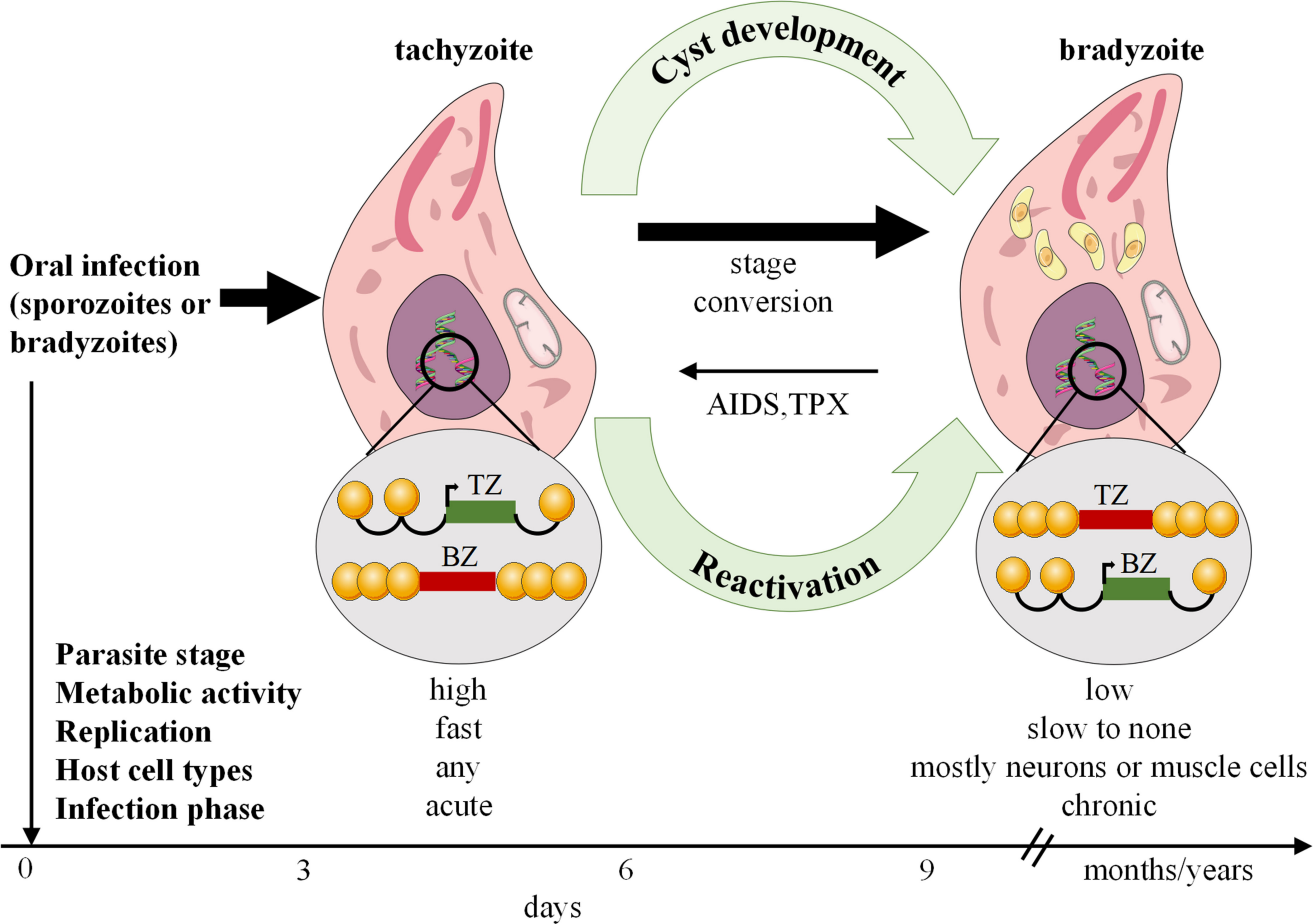
*Toxoplasma gondii* stage differentiation and its host (cell) microenvironment regulation. After infection, active tachyzoites convert to comparatively latent bradyzoites. The development of bradyzoite comprising tissue cysts for a long time is critical for transmitting the parasite to a new host. In immunocompromised such as AIDS or TPX patients, reconversion of bradyzoites to tachyzoites can occur and lead to serious disease. AIDS, Acquired immunodeficiency syndrome; TPX, Transplant recipients.

Toxoplasmosis is caused by *T. gondi*, an intracellular pathogen that affects roughly one-third of the global human population. It occurs in nature in a variety of forms, including oocytes, bradyzoites within latent tissue cysts, and actively replicating tachyzoites, which indicate active infection (32). *T. gondii* can invade nearly all nucleated cells of warm-blooded animals, existing within cells via the development of parasitophorous vacuoles (PV). Alongside PV formation, *T. gondii* employs various strategies to evade host immune defenses (33). Human transmission occurs through ingestion of contaminated food or water containing sporulated oocytes, consumption of undercooked meat containing latent cysts, vertical transmission from mother to child, or infected allografts during organ transplantation. On the other hand, acquisition via blood products or accidental exposure in laboratory settings is uncommon (34). Toxoplasmosis is a severe infection in immunocompromised patients, resulting in frequent reactivation of latent cysts in patients with chronic infection (35) (**Figure 2**). The disease is highly prevalence in AIDS patients, but its range has been changed and increased in other immunocompromised persons (36). Toxoplasmosis is particularly life-threatening in hematopoietic stem cell transplant or bone marrow patients (37).

### Current treatment options for toxoplasmosis

The preferred treatment for toxoplasmosis is a combination of sulphadiazine and Pyrimethamine (SDZ-PYR), which target the active stage of the infection (38). Additionally, alternative therapies are accessible, such as Pyrimethamine combined with certain antibiotics or the use of sulfamethoxazole-trimethoprim (ST) or atovaquone as monotherapy (34). If the patients cannot accept those molecules, an atovaquone 1500 mg combined with PYR can also be used, and SDZ combined with azithromycin 900 to 1200 mg/j can be prescribed (38). Some studies did not show an anti-parasitic relation between SDZ-PYR and PYR-clindamycin, therefore reinforcing the probability of selecting trimethoprim-sulfamethoxazole (39, 40). Trimethoprim-sulfamethoxazole is also the only successful combination for prophylaxis in patients who are at risk for *Toxoplasma* recurrence. For systemic treatment in immunocompromised patients, a combination of corticosteroids with anti-parasitic drugs was suggested for ocular toxoplasmosis (8, 41). However, comparative studies on this subject are rare. Due to a deficiency in clinical trials, SDZ-PYR is still the treatment choice when combined with corticosteroids (42). Recently, the pharmaceutical industries have been successfully manufacturing medicines using natural products to treat parasitic infections and discover their possible anti-parasitic properties (43).

### Omega-PUFAs efficacy in toxoplasmosis

Lipoxin A4, an eicosanoid mediator resultant from 5-lipoxygenase, has been shown to play a significant role in toxoplasmosis. It has been demonstrated that lipoxin A4, along with other arachidonic acid (AA) derivatives, promotes and enhances cyst burdens in tissues while reducing lethality from encephalitis. Previous studies have indicated a close association between AA, particularly lipoxin A4, and the suppression of cytokine generation such as interleukin 12 (IL-12) and IFN-γ (44). Notably, in a mouse model lacking lipoxin, a reduced number of *T. gondii* brain cysts were observed, accompanied by higher serum levels of IL-12 and IFN-γ (45). These findings suggest that the increased mortality in lipoxin-deficient mice resulted from cytokine-mediated tissue injury, despite effective parasite control. Antigen-presenting dendritic cells are crucial in controlling intracellular pathogens like *T. gondii* and certain viruses by producing IL-12. A study in mice revealed that lipoxin A4 analogs reduced IL-12 production by dendritic cells stimulated with *T. gondii* extract (45). This suggests that lipoxin production by pathogens may serve as a mechanism to modulate host immunity, potentially facilitating chronic infection by minimizing tissue damage. The differing roles of lipoxins in these infection models may be attributed to the dynamics of specific pathogen-host interactions. *T. gondii* replicates rapidly like *Mycobacterium tuberculosis*, which can trigger inflammatory reactions and subsequent immunopathology (46). Lipoxins demonstrate beneficial effects on the host, increasing survival by mitigating these inflammatory responses.

*T. gondii* has a considerable amount of unsaturated fatty acids, which are the structural membrane components that play a significant part in energy metabolism (47, 48). During replication, *T. gondii* requires a substantial quantity of lipids for membrane biogenesis, thus the propagation rates of tachyzoites depend on the production levels of fatty acids and the synthesis of new membranes. Additionally, the metabolism of fatty acids at the interface between the host and the parasite also influences *T. gondii* tachyzoites (49–51). Moreover, it has been demonstrated that the interface of fatty acid metabolism induces inflammatory cytokine production in the host and triggers calcium release from neutrophils, thereby facilitating the egress of parasites from infected host cells (52–54). However, Zhou et al. indicated that α-linolenic acid metabolism was significantly disrupted in mice infected with *T. gondii* (55). The administration of sulfadiazine sodium ameliorates the metabolomic perturbation in mice infected with *T. gondii*. These alterations in fatty acid metabolism may play a crucial role in facilitating *T. gondii* tachyzoites within cells. Hence, some authors have recommended the use of suitable nutritional supplementation, such as specific fatty acids (e.g., acyl-coenzyme A: diacylglycerol acyltransferase), in immunocompromised individuals to prevent chronic toxoplasmosis (56, 57).

### Possible mechanism of ω-PUFAs in *T. gondii* infection

**Figure 3:**
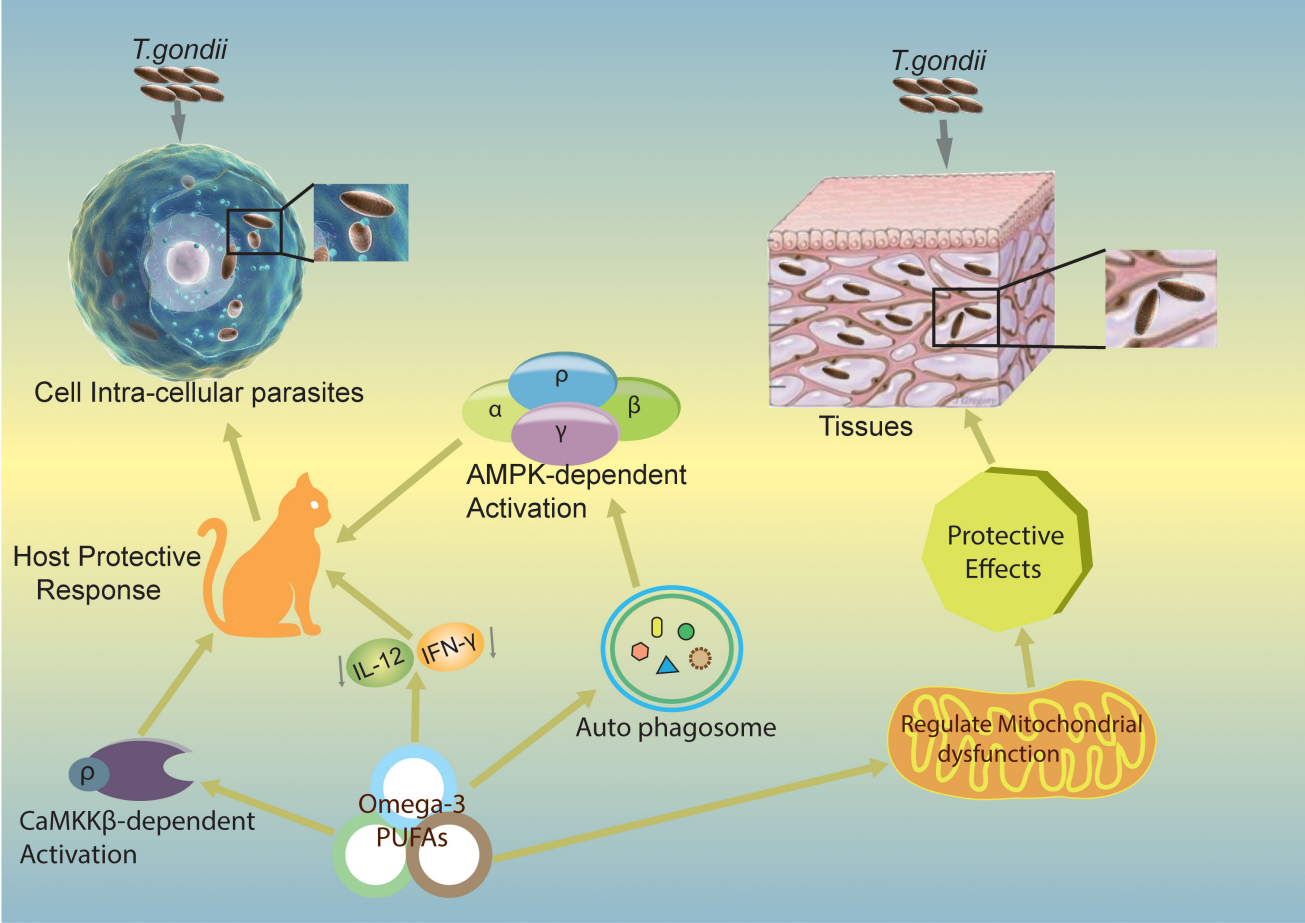
Mechanisms by which ω-3 PUFAs inhibit *T. gondii* infection. The figure shows potential mechanisms of action of ω-3 PUFAs in different pathways and proposes that ω-3 PUFAs are capable of producing a host-defensive response against *T. gondii* infection. Omega-3 induces CaMKKβ/AMPK-dependent autophagy signaling pathways activation (11) and decreases IL-12/IL-γ cytokines, which can lead to a produced host-defensive response against *T. gondii* infection. CaMKKβ, calmodulin-dependent protein kinase kinase β; AMPK, Adenosine monophosphate-activated protein kinase; IL-12, Interleukin 12; IL-γ, Interleukin gamma.

Numerous studies have elucidated the essential role of autophagy in removing intracellular pathogens through xenophagy. However, during *T. gondii* infection, two types of autophagy, canonical and non-canonical, are involved (58, 59). It has been suggested that ω-3 PUFAs play a significant role in regulating *T. gondii* infection both *in vivo* and *in vitro* (11). In primary macrophages, DHA significantly induces autophagy and mediates its activity, which is necessary to regulate and control *T. gondii* growth intracellularly (11). Moreover, the AMP-activated protein kinase (AMPK) activates the host-defense mechanism by regulating inflammatory responses and innate immunity to control infectious disease agents such as bacteria, viruses, and parasites (60). A recent study demonstrated that the calcium/calmodulin-dependent protein kinase kinase (CaMKK)/AMPK signaling pathways are required to eliminate *T. gondii* via CD40-induced autophagy (61). The two signaling pathways, CaMKKβ/AMPK-mediated autophagy, are essential for the host to control *T. gondii* infection. The proposed mechanism for this protection is depicted in **Figure 3**.

Studies have shown that nutrients play a crucial role in activating and maintaining immune homeostasis. Different nutrients, such as fatty acids, mediate both the acquired and innate immune systems through different pathways such as Toll-like receptor 4 (62). Similarly, various lipid components act as immune-activating factors and energy sources. During *T. gondii* infection, lipid biosynthesis occurs through salvage pathways, which play an essential role in intracellular pathogenesis and present promising chemotherapeutic targets in lipid synthesis and salvage pathways (49, 63). Moreover, a study recommended that the immunomodulatory functions of ω-3 PUFAs may be useful in inflammatory and infectious diseases (64).

### Malaria

Malaria is a mosquito-borne infectious disease caused by a protozoan parasite of the genus *Plasmodium* (65). The symptoms include fever, vomiting, fatigue, and headaches. In critical cases, malaria can cause seizures, yellow skin, coma, or even death (66). According to the World Health Organization, 228 million malarial cases occurred in 2018 (67).

### Current antimalarial drugs

The treatment of malaria and the choice of drug depends on the *Plasmodium* spp., drug resistance, geographical location, and disease severity (68). The complicated cases of malaria are treated with chloroquine 1000 mg (600 mg base), and it is the best choice of drug in most *Plasmodium* spp., except *P. falciparum*, which has become highly resistant in most cases (69). A combination of amodiaquine and artesunate, as well as atovaquone combined with proguanil (250 mg/100 mg), or an artemether 20 mg-lumefantrine 120 mg (twice daily for 3 days), can be used to treat chloroquine-resistant *P. falciparum* (69). During pregnancy, specified oral quinine and parenteral quinine are beneficial for severe malaria treatment. Doxycycline, mefloquine, primaquine, and atovaquone-proguanil are the drugs chosen for chemoprophylaxis (69). The list of antimalarial drugs and their mechanisms of action are shown in **Table 2**.

**Table 2:** Currently used antimalarial drugs.

Drug Name	Mechanisms of actions	References
Artemisinin and its derivatives	Haem-generate free radicals, damage parasite survival protein	(70, 71)
Amodiaquine	Complexation with haem and stop hemozoin development	(72)
Piperaquine	Inhibiting haem detoxification, collecting in the digestive vacuole	(73)
Lumefantrine	Constrains protein and nucleic acid synthesis inhibits the development of β-haematin	(73, 74)
Proguanil	Acts as a DHFR inhibitor and disrupts the synthesis of deoxy thymidylate	(75)
Atovaquone	Blocks mitochondrial electron transport acts as cytochrome *bc_1_* complex inhibitor	(76)
Pyrimethamine	Target the parasite folate biosynthesis pathway	(77, 78)
Sulfadoxine	Target the parasite folate biosynthesis pathway	(78)
Pyronaridine	Inhibit β-haematin formation	(79, 80)
Tafenoquine	Prodrug metabolized to the active quinone	(81)

### Omega-PUFAs efficacy in malaria

The effects of ω-PUFAs on malarial causative agents such as *P. berghei* or *P. falciparum* are the most reliable and easily understood. Studies have shown that indomethacin did not affect cerebral malaria or parasitemia development; however, ω-3 and ω-6 PUFAs acted directly on the pathogen and constrained parasitemia both *in vivo* and *in vitro* (82). This inhibition mechanism depends on the concentration of fatty acids, their unsaturation, and chain length. A mouse model infected with *P. berghei* and treated for 4 days with fish oils rich in PUFAs showed suppressive effects against the parasite (82, 83). In another study, the authors confirmed that human phospholipids-derived PUFAs exert a strong *in vitro* anti-*plasmodium* action mainly by hydrolyzing lipoproteins from plasma, thus releasing PUFAs that are toxic to the parasite (84).

The addition of antioxidants or reactive oxygen species decreased ω-3 PUFA’s capability to kill *P. falciparum*, but the oxidized fatty acid’s addition improved their capability to destroy the parasite (85). A study conducted by Blok et al. showed that there was no requirement of vitamin E deficiency for ω-PUFAs to distress antimalarial resistance (82, 86). Fish oil consumption significantly reduced hepatic and plasma vitamin E. However, it did not alter the immune cell vitamin E status (82). These results strongly proposed that ω-PUFAs increased host survival rate without showing any changes in the host immune system and exerted direct cytotoxic action on *Plasmodium* spp.

### Coccidiosis

Coccidia is a group of obligate intracellular protozoan parasites that fall under the class Conoidasida within the phylum Apicomplexa. Two notable genera within this group are *Eimeria*, characterized by sporulated oocysts containing four sporocysts, and *Isospora*, which has sporulated oocysts with two sporocysts. These genera include a wide variety of species capable of infecting birds, mammals, and reptiles, but it is essential to emphasize that most of these species demonstrate a strong preference for specific host species. Infections caused by these parasites are widespread across the globe (87).

### Existing strategies to control coccidiosis

Although good animal husbandry can reduce coccidian parasite transmission, some additional measures are important to lower the risk of infection (88). Currently, some coccidiocidal drugs are used as regular prevention and therapies for the disease and inhibit the growth and replication of coccidial populations. When anticoccidial drugs are withdrawn, the infective oocysts contaminate the environment again and continue their life cycle (89).

The combination of several drugs shows more efficiency in controlling coccidiosis. In this regard, since the 1960s, sulfonamide has been used as a potent drug for the control of *Eimeria* spp. infection, many chemical synthetic drugs, and ionophores compounds were developed or found using coccidiostatic or coccidiocidal agents. However, new potent molecules urgently need to be found and used to control the disease because of the increasing drug resistance (89). To stop drug resistance, the rotation and shuttle systems are urgently needed, and drug type is used to switch after one or several (90). The attenuated and un-attenuated vaccines are also used aiming to control coccidiosis; however, the effectiveness of vaccines is based on oocysts and immunity (91). The practice of live un-attenuated vaccines, such as Inovocox, Coccivac, etc., is inadequate because they induce the risk of live parasites (92). However, live attenuated vaccines such as Paracox and HatchPak CocciIII reduce disease risk, and the intestinal segments of birds are less damaged (93). To fully combat the infection, there is still a required fully effective anticoccidial drug or vaccine according to the species-specific nature of immunity. Moreover, the anticoccidial drug resistance detected in birds worldwide has shown that natural products with efficient anti-coccidial activity will be more efficient (94).

### Omega-PUFAs efficacy in coccidiosis

The combination of ω-PUFAs in the diet of chicken challenged with *Eimeria tenella* decreases the contrary effects on development and gut lesion scores. The inclusion of ω-3 PUFAs can help to control *E. tenella* infection and improve the resistance of poultry to the pathogen. These effects in broiler chickens were investigated compared to the earlier findings, which showed ω-3 PUFAs afford some protective effects against malaria (95). The carotenoid level in total plasma reduced the infection of coccidia (96), while free radicals such as peroxynitrite levels increased (97). Researchers assumed that DHA, EPA, and LA generate oxidative stress that stops coccidia development by being incorporated into the membranes of the parasite. There, they are extremely prone to oxidation by leucocyte-free radical manufacturing, which affects the coccidian (97). In another study, corn oil was compared with fish oil in *E. tenella-infected* chickens, in which fish oil successfully reduced lesion score, TNF-α, and inflammatory process (98).

### Chagas disease

Chagas disease (CD), commonly known as American trypanosomiasis, is a potentially fatal infection caused by the protozoan parasite *Trypanosoma cruzi*. *T. cruzi* is a parasitic protozoan spread to mammalian hosts by blood-sucking bugs called triatomine, and in humans and animals, it leads to anemia, parasitemia, and hepatosplenomegaly (99). The mortality rate during acute *T. cruzi* infection is rare, but its occurrence results severe decline in leukocytes and circulating platelets (100, 101). Everything that disturbs host immune responses, such as AIDS, aging, chemotherapy, or antirejection treatment resulting in the transplantation of an organ, can endorse a recurrence of this dormant infection with successive morbidity (102, 103).

### Trypanocidal agents

Different compounds, such as mercury chloride, arsenic, emetic tartrate, fuchsine, and even the antiseptic gentian violet, were tried to treat the disease. However, the results were unsatisfactory (102, 103). Later in the 1960s, numerous novel compounds, such as the nitrofurans that act as an antimicrobial agent, were tested in which nitrofurfurylidene, known as nifurtimox (RS)-3-methyl-N-[(1E)-(5- nitro-2-furyl) methylene] thiomorpholin-4-amine 1, 1-dioxide) (NF), showed satisfactory results (104).

Nevertheless, the first promising drug for CD treatment was Nifurtimox (NFX), which was used by Packchanian (105), and its clinical trials were started in South America in 1965. In adult, chronically infected patients, the effectiveness of NFX was low, with a rate of 7-8%, but in young children aged < 14 years, this drug showed a higher cure rate of 85.7% (106). The most common adverse effects of NFX are weight loss, anorexia, gastrointestinal symptoms, vomiting, etc. Another drug, benznidazole (BZ), was shown to be useful for the treatment of CD (104). BZ was shown to be less toxic than NFX. However, their efficacy for treating CD is almost similar. Age is a significant factor for BZ effectiveness as well. In children around 6-12 years of age, BZ showed a higher cured efficacy of 56-62% (104). The mechanisms of action of both BZ and NFX are still unclear; however, it has been shown that BZ acts as a reductive stress molecule that affects the trypanothione metabolism of *T. cruzi* (107). This drug can also induce IFN-γ, raise trypanosomal death, and improve phagocytosis via inhibiting *T. cruzi* NADH-fumarate reductase (104). NFX produces extremely toxic oxygen metabolites that render *T. cruzi* to limited reduction products of oxygen, typically hydrogen peroxide (104). The complete list of drugs and their status for the treatment of CD are shown in **Table 3**.

**Table 3:** Drugs and their current status for the treatment of Chagas disease.

Drug	*In vitro* assay	*In vivo* assay	Phase I	Phase II	Phase III	Approval
BZ	**√**	**√**	**√**	**√**	**√**	**√**
NFX	**√**	**√**	**√**	**√**	**√**	**√**
Se	**√**	**√**	**√**	**√**	In progress	**×**
ALBA	**√**	**√**	**√**	**-**	**-**	**×**
ALOPU	**√**	**√**	**√**	**×**	**-**	**×**
FENARI	**√**	**√**	Planned	**-**	**-**	**×**
AMIO	**√**	**√**	**√**	In progress	**-**	**×**
SCYX-7158	**√**	**√**	In progress	**-**	**-**	**×**
POSA	**√**	**√**	**√**	**√**	**-**	**×**
KETO	**√**	**√**	**√**	**×**	**-**	**×**
RAVU	**√**	**√**	**√**	**√**	**-**	**×**
ILMOFO	**√**	**-**	**-**	**-**	**-**	**×**
VORI	**√**	**√**	**√**	**-**	**-**	**×**

BZ, Benznidazole; NFX, Nifurtimox; Se, Selenium; ALBA, Albaconazole; ALOPU, Allopurinol; FENARI, Fenarimol; AMIO, Amiodarone; SCYX-7158, Oxaborole; POSA, Posaconazole; KETO, Ketoconazole; RAVU, Ravuconazole; ILMOFO, Imofosine; VORI, Voriconazole.√ mean Done, ⨯ mean interrupted, - mean haven't pass to next phase.

### Omega-PUFAs and *T. cruzi*

**Figure 4:**
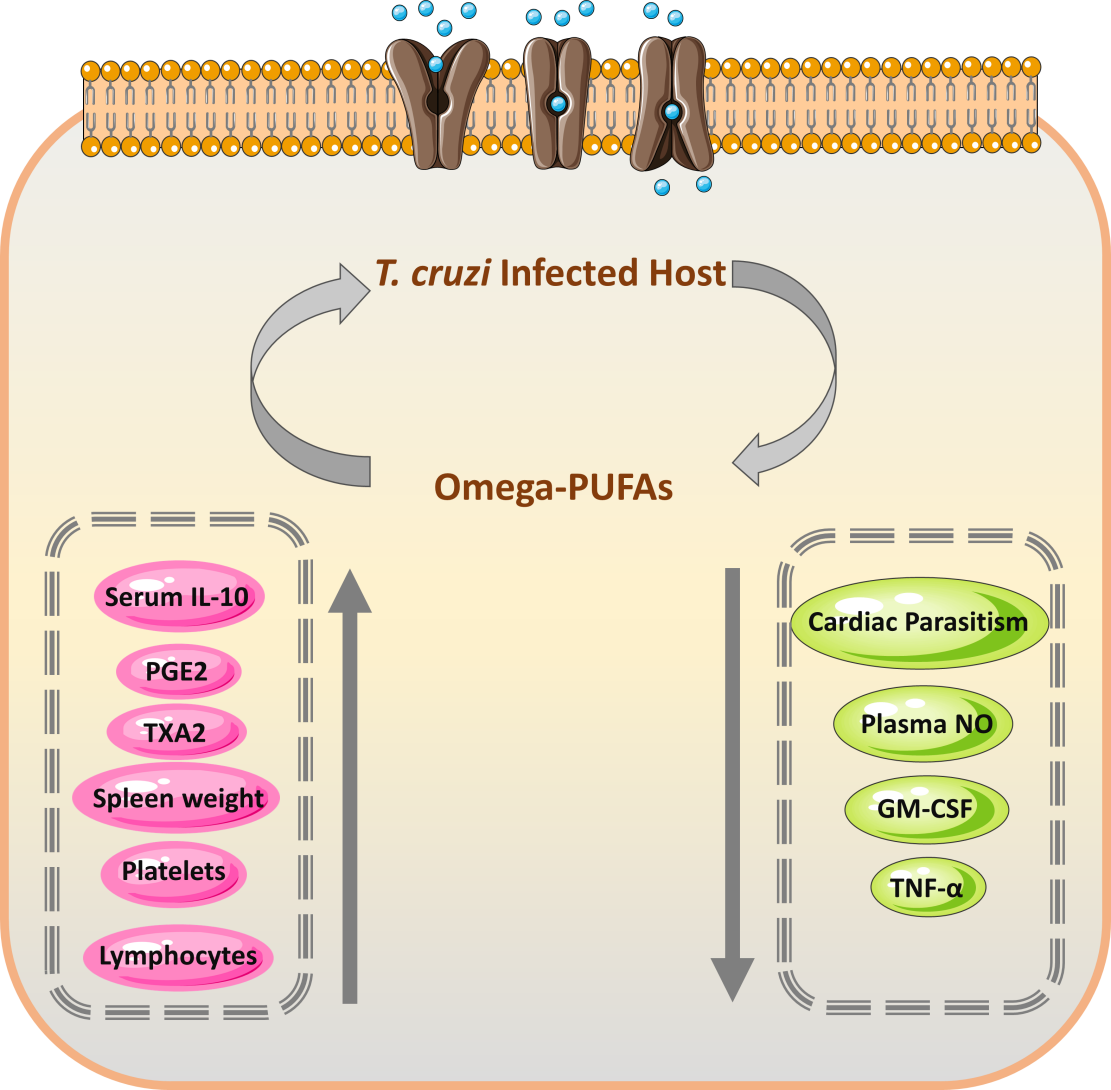
Mechanism and efficacy of omega-PUFAs supplementation on *T. cruzi* infection. During the acute form of *T. cruzi* infection, the increased level of prostaglandin E2 (PGE2) results in temporary immunosuppression, which leads to decreased production of TNF-α by the host (112). The immune cells from the omega-PUFAs diet lowered PGE2 amounts and consequently increased the level of TNF-α leading to decreased parasitemia (111). PGE2, Prostaglandin E2; TXA_2_, thromboxane A2; GM-CSF, Granulocyte-macrophage colony-stimulating factor; TNF-α, Tumor necrosis factor-alpha.

Nutrient supplements and some dietary fats are known to have influenced the function of the immune system (108). They can affect both cellular (T cells, B cells, and natural killer (NK) cells that may modulate the production and activity of cytokines) and innate immunity, influencing survival during infections. These supplements may contribute to the protection against parasitic infections including *T. cruzi*. Previous studies demonstrated that ω3 and ω6 PUFAs impacted cellular and innate immunity (109, 110). These PUFAs also affected the survival of mice following an experimental *T. cruzi* infection and showed fewer indications during the acute phase of this parasitic infection (111). The mechanisms for this protection are shown in **Figure 4**. Recently, many studies stated the trypanocidal actions of some natural medicine or their extracts (107, 113). In some Asian countries such as Vietnam and Uzbekistan, there was much use of plant extracts and their constituents that showed trypanocidal activities against *T. cruzi* (114).

### Possible drawbacks related to the use of ω-PUFAs

While omega polyunsaturated fatty acid ω-PUFAs offer numerous health benefits, there are also potential drawbacks associated with their uses. Due to the use of ω-PUFAs, there is the possibility of digestive issues, allergic reactions, and bleeding risk. The possibility that enhances these drawbacks is discussed below.

### Inadequate associated studies and clinical trials

In China, ω-3 and ω-6 PUFAs have been widely studied in treating several kinds of cardiovascular diseases, cancer, inflammations, physical damage, etc., while research on the anti-parasitic functions is seldom reported. Moreover, researchers have obtained information on ω-3 and ω-6 PUFAs as described above, but the clinical studies trials are still deficient. These studies are critical to validate the anti-parasitic activities of ω-3 and ω-6 PUFAs.

### Dosage

Until now, the recommended dosage of PUFAs has also been an issue in clinical trials. Previously, in cancer studies the researchers used a higher dose level of EPA + DHA ranging from 2.0 to 8.0 g/kg body weight/day in mice (115). Subsequently, the interspecies alteration lower dosage for animals also seemed higher for humans (about 10–20 g/day in 70 kg per person) (116). In human interventional experimental trials, the protective effects of ω-3 PUFAs were observed with a 2.0 g/day dosage (consistent with 0.03 g/kg body weight in a 70 kg person) (117, 118). The best preventive effects of these PUFAs were accompanied by increasing the dosage of tissue lipids or plasma in both humans and animals (115). Excessive PUFAs could lead to unintentional health problems in certain conditions and nutritional standards based on the best available evidence still need to be established.

### Administration route

Different routes, such as oral, inhalational, intravenous, and topical, are used to administer substances into the body. The administration route for an active component to the targeted place is significantly important. Experimental trials have shown the effects of ALA on numerous conditions in humans, such as lipid metabolism ailments and cardiovascular diseases, but many of them work on the oral direction as the entry route. For ω-PUFAs, strong solubility and good delivery routes are important because poor solubility and delivery may have influenced the bioavailability of PUFAs (119, 120). Thus, additional human and animal studies are particularly needed to precisely display the administration route of ω-PUFAs with the best delivery ability.

## Conclusion and future perspectives

The treatment options for parasitic infections are not sufficiently effective due to significant side effects, necessitating the urgent development of novel therapeutic choices. We propose that ω-3 and ω-6 PUFAs may be potentially effective in ameliorating some parasitic infections and associated abnormal conditions. These PUFAs have been proven to play a key role in modulating immune responses and activating certain signaling pathways in various parasitic infections. The evidence elucidated in the current review suggests that further experiments would be essential to combine these PUFAs with other drug candidates to assess the clinical impact of ω-3 and ω-6 in parasitic infections. Finally, we recommend that future studies focus on:

1.Understanding of the main pathway/s responsible for the activities of ω-3 and ω-6 PUFAs in parasitic infections.
2.Determining the most effective doses for the beneficial role of ω-PUFAs in various parasitic diseases.
3.Exploring the possible effects of ω-3 and ω-6 on emerging treatments, such as microRNAs by targeting signaling pathways and or expression profiles.
4.Examining the possible interactions of ω-3 or ω-6 with well-known anti-parasitic drugs, as well as nutritional supplements.

## Author contributions

SUR: Writing – review & editing, Writing – original draft, Software. TW: Writing – review & editing. AQ: Writing – review & editing, Investigation. SN: Writing – review & editing. HU: Writing – review & editing. CC: Writing – review & editing, Validation, Funding acquisition.
